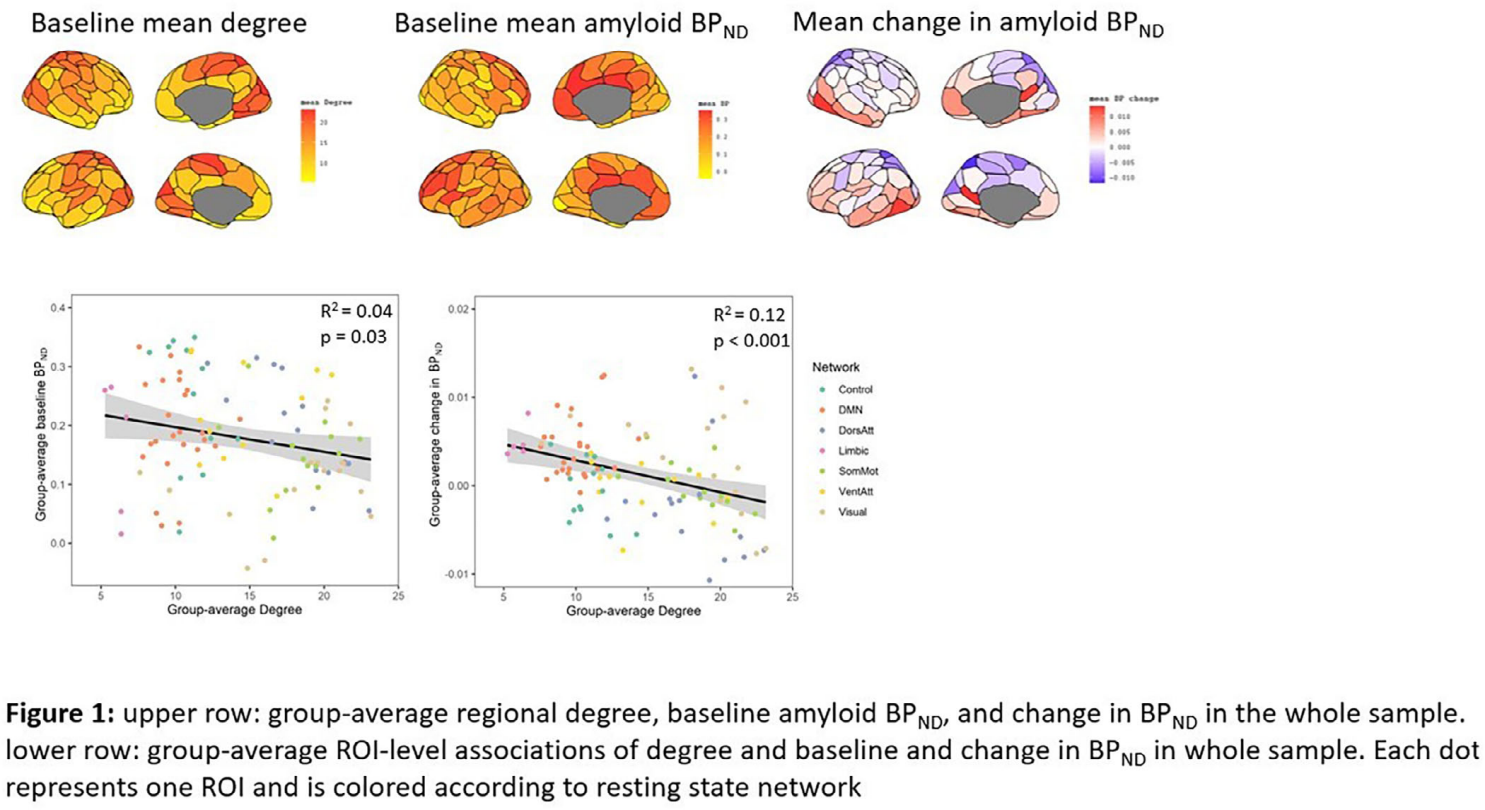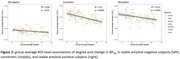# Functional Connectivity and Longitudinal Changes in Amyloid Pathology in Cognitively Healthy Older Adults

**DOI:** 10.1002/alz.088062

**Published:** 2025-01-09

**Authors:** Mara ten Kate, Luigi Lorenzini, Betty M. Tijms, Alle Meije Wink, Elles Konijnenberg, Jori Tomassen, Sophie M. van der Landen, Eco J.C. de Geus, Emma M. Coomans, Lyduine E. Collij, Philip Scheltens, Elsmarieke van de Giessen, Pieter Jelle Visser, Anouk den Braber, Frederik Barkhof

**Affiliations:** ^1^ Department of Radiology & Nuclear Medicine, Vrije Universiteit Amsterdam, Amsterdam UMC, location VUmc, Amsterdam Netherlands; ^2^ Department of Radiology and Nuclear Medicine, Amsterdam Neuroscience, Vrije Universiteit Amsterdam, Amsterdam UMC, Amsterdam Netherlands; ^3^ Alzheimer Center Amsterdam, Department of Neurology, Amsterdam Neuroscience, Vrije Universiteit Amsterdam, Amsterdam UMC, Amsterdam Netherlands; ^4^ Alzheimer Center Amsterdam, Neurology, Vrije Universiteit Amsterdam, Amsterdam UMC, Amsterdam, Noord‐Holland Netherlands; ^5^ Department of Biological Psychology, Vrije Universiteit Amsterdam, Amsterdam Netherlands; ^6^ Department of Radiology and Nuclear Medicine, Vrije Universiteit Amsterdam, Amsterdam University Medical Center, location VUmc, Amsterdam Netherlands; ^7^ Amsterdam Neuroscience, Neurodegeneration, Amsterdam Netherlands; ^8^ Department of Radiology & Nuclear Medicine, Amsterdam UMC, Amsterdam Netherlands; ^9^ Alzheimer Center and Department of Neurology, Amsterdam Neuroscience Campus, VU University Medical Center, Amsterdam Netherlands; ^10^ Amsterdam UMC, Amsterdam Netherlands; ^11^ Department of Radiology and Nuclear Medicine, Vrije Universiteit Amsterdam, Amsterdam UMC, Amsterdam Netherlands

## Abstract

**Background:**

Accumulation of amyloid beta in the brain is one of the first events in Alzheimer’s disease (AD) and starts decades before symptoms arise. It has been hypothesized that brain areas with higher levels of neuronal activation (‘hubs’) are more prone to amyloid deposition. In this study, we examined the regional relationship between cortical hubs and longitudinal changes in amyloid pathology in a sample of cognitively healthy older subjects.

**Method:**

Participants were included from the EMIF‐AD PreclinAD cohort, a longitudinal study in cognitively healthy older monozygotic twins. Standard resting state functional MRI pre‐processing was performed with fMRIprep. Individual connectivity matrices were generated as the pairwise timeseries correlation between 100 regions in the Schaeffer atlas, and binarized using a proportional threshold of 0.25. Regional degree, the number of connections of each region, was taken as a continuous measure of functional 'hubness'. Degree was computed within the 100 individual regions, and averaged within 7 resting state networks. Dynamic [^18^F]Flutemetamol PET scans were used to visually assess amyloid status, and to quantify global and regional binding potential (BP_ND_) at baseline and follow‐up (mean 4.4±0.5 years). We first assessed whether baseline regional degree could predict change in amyloid status using GEE to account for familial clustering. Next, we performed ROI‐wise analyses, assessing the relationship between group‐average regional values of degree and quantitative BP_ND_ (baseline and change) in the whole sample and according to amyloid subgroups (negative stable, converter, positive stable).

**Result:**

We included 124 subjects (age 68.3±6.4, female 54%). In baseline amyloid visually negative subjects (n=111), a lower mean degree in the default mode network (β=‐0.26, p=0.008) was associated with higher chance of conversion from negative to positive amyloid status. In the ROI‐wise analyses, a lower degree was associated with higher baseline BP_ND_ and larger rate of increase over time (Figure 1). This relationship was most pronounced in the converter group (Figure 2).

**Conclusion:**

We found that in cognitively healthy individuals, decrease rather than increase in functional hubness is associated with amyloid load and accumulation. Possibly in this population of older adults, functional hubs are already affected by ageing, or early pathology.